# Traditional Chinese medicine as potential adjuvants for tumor vaccines: a review of types, mechanisms, and forms

**DOI:** 10.3389/fimmu.2025.1661694

**Published:** 2025-12-10

**Authors:** Yongjia Cui, Lutian Gong, Lei Chang, Wenping Lu, Siqing Zhao, Zhili Zhuo, Xiangyang Zhang

**Affiliations:** 1 Guang’anmen Hospital, China Academy of Chinese Medical Sciences, Beijing, China; 2 Postdoctoral Research Station, China Academy of Chinese Medical Sciences, Beijing, China; 3 Beijing University of Chinese Medicine, Beijing, China

**Keywords:** traditional Chinese medicine, adjuvant, tumor vaccines, cold tumors, immunotherapy

## Abstract

Therapeutic cancer vaccines are increasingly recognized as a highly promising approach for tumor treatment; however, their clinical efficacy critically depends on the rational design of vaccine adjuvants. Natural compounds derived from Traditional Chinese Medicine (TCM) have emerged as attractive candidates for next-generation tumor vaccine adjuvants. In this review, we systematically summarize the chemical constituents of TCM-derived adjuvants reported in current research and categorize their mechanisms of action into four functional modalities: (i) immunostimulatory adjuvants that activate innate immune pathways; (ii) delivery-enhancing systems that improve antigen stability and facilitate targeting to antigen-presenting cells; (iii) integrated platforms that combine both immunostimulatory and delivery-enhancing functions; and (iv) other mechanisms involving non-canonical immunomodulatory activities. We further discuss current challenges in standardization, regulatory approval, and clinical translation, offering a roadmap for harnessing the potential of TCM in the rational design of cancer vaccines.

## 1 Introduction

Immunotherapy has significantly advanced cancer treatment and is now considered a leading therapeutic strategy. Immunotherapy eliminates cancer cells by activating the body’s immune system rather than directly killing cancer cells (1, 2). According to the infiltration of T lymphocytes in the tumor microenvironment, tumors can be divided into “hot” (immune-inflamed) phenotype and “cold” (immune-desert) phenotype. Hot tumors respond better to immune checkpoint inhibitors (such as PD-1/PD-L1 inhibitors). In contrast, “cold” (immune-desert) phenotype, including ovarian cancer, refers to tumors with less immune cell infiltration and low immune activity, and usually respond poorly to immunotherapy (3). Therefore, exploring new immunotherapy methods (including therapeutic tumor vaccines) to stimulate T cell production has attracted increasing attention (4). So far, encouraging efficacy has been observed in clinical trials of tumor vaccines to treat tumors. The U.S. Food and Drug Administration (FDA) has approved three therapeutic tumor vaccines, namely, Sipuleucel-T, Bacillus Calmette-Guerin (BCG), and Talimogene laherparepvec (TVEC) (5). These approved vaccines demonstrate anti-tumor activity and the potential to prolong survival. The inactivated and live-attenuated vaccines previously used were effective but unsafe. The successful deployment of mRNA vaccines against COVID-19 has stimulated people’s interest in genetic vaccination for cancer treatment (6). Therefore, using antigens with good properties in vaccines, such as nucleic acids, purified proteins, or peptide sequences, has become a new development trend. Still, their low antigen utilization rate has hindered their clinical development (7). Improving the antigenicity of tumor vaccines is the key to the effectiveness of tumor vaccines.

An adjuvant is a substance that can be added to a vaccine to stimulate and enhance the intensity and durability of the immune response (8). Adjuvants can be divided into immunostimulants and delivery systems (9). Immunostimulants activate specific signaling pathways to promote the activation and maturation of antigen-presenting cells, such as the toll-like receptors (TLR) and cGAS-STING pathways. Mature antigen-presenting cells terminate their antigen-phagocytic activity, enhance their ability to present antigens, and express high levels of co-stimulatory signals and cytokines, which lead to the initiation and enhancement of adaptive immune responses. The delivery system mainly protects and targets antigens to antigen-presenting cells (APCs). The FDA has approved the development of adjuvant delivery systems, including aluminum, MF59, AS04, AS03, AS01, and CpG ODN 1018 (10). However, there is still a need to explore and develop new adjuvants to improve the efficacy of tumor vaccines.

In recent years, Traditional Chinese Medicine (TCM) has demonstrated immunomodulatory activity and can improve humoral and cellular immunity, attracting much attention (11–14). Polysaccharides, saponins, and other components derived from TCM have immunomodulatory characteristics, biocompatibility, biodegradability, low toxicity, and safety. A novel TCM-based nano-formulated *in situ* tumor vaccine has been developed, which employs tumor fragments from minimally invasive cryoablation as autologous antigens to target lymph nodes, elicit tumor antigen-specific immunity, and generate a systemic anticancer response following local administration—demonstrating significant translational potential (15). Some reviews have focused on the potential of polysaccharides and saponins from TCM as adjuvants (16, 17). Still, the study of another ingredient from TCM in adjuvants has not yet been evident. In addition, we noticed that vesicles or spore powders derived from TCM can be used as vaccine delivery systems.

**Figure 1:**
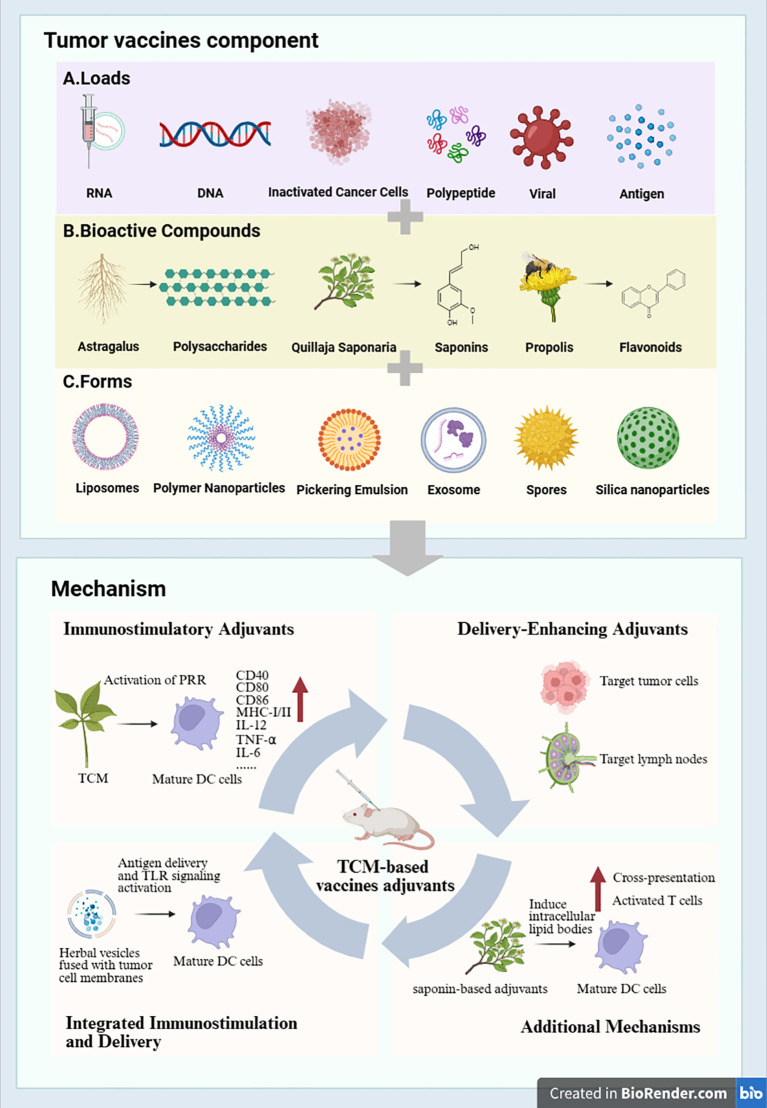
Components, formulations, and mechanisms of tumor vaccines incorporating traditional Chinese medicine (TCM) as adjuvants. **(A)** Components of tumor vaccines include various loads such as RNA, DNA, inactivated cancer cells, polypeptides, viral components, and antigens. **(B)** Bioactive compounds—such as astragalus polysaccharides, quillaja saponins, and propolis flavonoids—serve as immunostimulatory or delivery-enhancing agents. **(C)** Various formulations are used to enhance the efficacy of TCM-based adjuvants, including liposomes, polymer nanoparticles, Pickering emulsions, exosomes, spores, and silica nanoparticles. DCs, dendritic cells; PRRs, pattern recognition receptors; TLR, Toll-like receptor. (Figure was created using https://app.biorender.com/).

While previous reviews have addressed individual classes of TCM-derived adjuvants (e.g., polysaccharides or saponins), a systematic integration of their immunomodulatory mechanisms, delivery platforms, and translational challenges—particularly in the context of cold tumors—is still lacking. Here, we bridge this gap by providing a comprehensive analysis of TCM-based adjuvants, summarizing both their key bioactive compounds (e.g., polysaccharides, saponins, and flavonoids) and their immunostimulatory mechanisms, while also covering emerging formulation strategies—with an emphasis on their potential to convert immunologically cold tumors into responsive ones (**Figure 1**).

## 2 TCM-derived compounds as adjuvants for tumor vaccines

### 2.1 Key bioactive constituents of TCM-derived vaccine adjuvants

#### 2.1.1 Polysaccharides

Polysaccharides (PS) are natural polymers produced by plant cell metabolism, typically composed of monosaccharide units linked by glycosidic bonds. Five groups of polysaccharides can be distinguished based on their sources: bacterial, fungal, plant, lichen, and algal polysaccharides, as well as animal polysaccharides (18). The variety of glycosidic bond types and monosaccharide species gives polysaccharides a range of biological activity characteristics. Plant polysaccharides have demonstrated notable immunomodulatory properties because of their rich biological information and varied skeletal topologies (19). In addition to promoting cytokine release and antibody production, polysaccharides are natural immunomodulators that stimulate immune cells, including T/B lymphocytes, macrophages, and NK cells. They can also activate the complement system and regulate signaling pathways connected to tumors (20). Specifically, polysaccharides are crucial for accelerating DCs maturation, activation, and macrophage polarization. The primary mechanism entails the control of signaling pathways, including PI3K-Akt, TLR, NF-κB, MAPK, and JAK-STAT (21).

Astragalus polysaccharide, angelica polysaccharide, atractylodes polysaccharide, and ganoderma polysaccharide are examples of polysaccharides from TCM that have been demonstrated to be effective adjuvants for tumor vaccines that can boost immune efficiency (22–25). Here, we present several novel polysaccharides that may be used as adjuvants in tumor vaccines. For example, SMPD-2, a polysaccharide derived from Salvia miltiorrhiza, significantly increased the levels of IL-1 and IFN-γ secreted in the supernatant of mouse peritoneal macrophages *in vitro*. Moreover, *in vivo* tests demonstrated that SMPD-2 might stimulate a potent, targeted humoral immune response and encourage DC activation in draining lymph nodes. Importantly, As a vaccine adjuvant, SMPD-2 promotes the activation of CD4^+^ and CD8^+^ T cells, enhances the CTLs response, and thereby amplifies antitumor immunity (26). DsCE-I, a polysaccharide component isolated from Dioscorea batatas, had strong immune adjuvant efficacy when added to a mouse model to prevent B16-hgp100 melanoma utilizing DNA vaccination. DsCE-I can activate NF-κB. This demonstrates that DsCE-I has a wide range of potential uses in controlling immune responses and preventing the formation of tumors (27). When combined with DCs vaccinations, the purslane-derived polysaccharide POL-P3b can dramatically reduce tumor growth in mice with breast cancer. The TLR4/MyD88/NF-κB signaling pathway primarily activates and matures DC, boosting the immune system and preventing tumor growth by encouraging cell death. This approach may help address the limited efficacy and low antigen immunogenicity associated with DC vaccines (28). The rhizome of Bletilla striata contains a natural polysaccharide called PRBS, which has demonstrated promise as a multipurpose immune adjuvant. Numerous immune cells, including macrophages, B cells, and DCs, can be activated by PRBS when it co-assembles various antigens. Its favorable safety profile and biocompatibility support its use as a platform for self-adjuvanting nanovaccines, facilitating further development of anti-tumor vaccines (29). In conclusion, polysaccharides used in TCM typically possess the following traits: a significant molecular weight, a complex spatial structure, a wide distribution in nature, and multi-target immunomodulatory actions. As technologies like molecular modification and nanocarriers have advanced recently, there has been a steady increase in the study and use of polysaccharides as adjuvants in tumor vaccines. Enhancing the chemical properties and targeting capacity of TCM-derived polysaccharides may expand their utility in cancer immunotherapy.

#### 2.1.2 Saponins

Because of their distinct chemical structure and biological action, saponin natural compounds have garnered much interest in vaccine adjuvant research. Triterpenoid saponins and their derivatives, derived from Quillaja saponaria, its primary constituent, have low-dose and high-efficiency adjuvant activity and the capacity to modulate the immune system (30). The distinct hydrophilic-hydrophobic-hydrophilic polar sections of saponin molecules can influence the type of immunological response and are intimately linked to their adjuvant function. Because of this characteristic, saponins are frequently found in liposomes, nanoparticles, and other forms of nanoparticles for use in tumor immunotherapy, complementing Toll-like receptor 4 (TLR4) agonists and boosting helper cell and cytotoxic T cell responses (31). As the active components of saponins, triterpenoid saponins can further elicit innate and acquired immune responses by causing T cells to generate IL-2, which in turn triggers the production of secondary cytokines, including TNF, IL-1, and IL-6 (32). Astragaloside VII (AST VII) and its derivatives, which were isolated from Astragalus plants, for instance, caused DC cell maturation and T cell activation, increased the secretion of IL-1β and IL-12, and significantly improved the pro-inflammatory response in mouse bone marrow-derived DCs treated with LPS (33). Because they possess a slight hemolytic activity, other saponin components, like saponins (PCS) derived from Pulsatilla chinensis’ root, have demonstrated potent vaccine adjuvant effectiveness in *in vivo* tests. In addition to stimulating spleen cell proliferation, high-dose PCS raises serum levels of specific antibodies (IgG, IgG1, and IgG2a) and cytokines, including IL-2 and TNF-α. PCS is considered a safe and efficient vaccination adjuvant that can simultaneously control Th1 and Th2 helper T cell responses, improving adaptive immune responses (34).

Additionally, the study demonstrated that saponin adjuvants enhanced DCs’ capacity to present antigens cross-presentation and markedly stimulated CD8+ T cells by activating the PERK pathway and promoting the creation of liposomes (35). This opinion was further supported by later research on the reaction of DC subsets to saponin adjuvants conducted by Nataschja I. Ho et al. (36). This characteristic allows for the creation of novel adjuvants to enhance cancer vaccinations (37). For instance, Basic fibroblast growth factor (bFGF), an angiogenic factor implicated in tumor neovascularization and cancer cell metastasis, was complexed with saponin-containing liposomes to form a lipo-saponins/bFGF complex. Immunity targeting bFGF may represent an effective strategy for cancer immunotherapy. In BALB/c mouse models, this complex suppressed tumor-associated angiogenesis while simultaneously eliciting robust antibody responses (38). Furthermore, the antigen OVA and the novel nano-encapsulated plant immunostimulatory molecules [primarily a new nano-adjuvant with Saponin D (SND)] can boost the immune system and have anti-tumor effects in the new anti-tumor vaccine (39).

In vaccine development, tumor vaccines must stimulate anti-tumor CTL responses by improving antigen cross-presentation by DCs. In contrast, microbial vaccines primarily elicit antibody responses through antigens and non-microbial adjuvants. According to studies, tumor ablation alone can only offer a limited degree of immune protection. However, when paired with adjuvants based on saponins, it can significantly improve DC’s capacity to capture and activate antigens and raise the degree of antigen cross-presentation, which triggers tumor-specific CTL responses and results in long-lasting tumor immune protection. This approach is appropriate for various human and veterinary tumor therapy situations. It can be employed as an effective *in situ* DC vaccination strategy without requiring prior knowledge of tumor antigens (40).

#### 2.1.3 Flavonoids

Plants include a large family of polyphenolic chemicals called flavonoids. Isoflavones, flavanols, flavanones, flavonoids, and anthocyanidins are the six significant subclasses (41). Currently, luteolin, hesperetin(HES), procyanidin(PC), and other flavonoid compounds are commonly utilized as vaccine adjuvants. Flavonoids have been shown to boost the immune response in antiviral, anti-infection, and other therapies. Flavonoids have been reported to enhance immune responses in antiviral and anti-infective contexts (42). The application of flavonoids as tumor vaccine adjuvants is mainly concentrated in melanoma, and there is less research on other tumors. For instance, natural propolis and other TCM frequently contain the flavonoid chrysin (CHR). By triggering the IL12-STAT4 signaling pathway, CHR may improve Th1 cell activity and boost the anti-tumor response of CTL *in vivo*. In the B16F10 mice model, CHR activated both *in vivo* and *in vitro* APCs. Furthermore, B16F10 tumor-bearing mice survival rate was markedly increased when CD8+ T cells from immune mice were transferred into them. These findings indicate that CHR can function as a vaccine adjuvant by enhancing APC activation and CD8^+^ T cell–mediated antitumor immunity (43). The TCM has utilized luteolin to treat cancer, inflammation, and high blood pressure. Its antioxidant and anti-inflammatory qualities are linked to its anti-cancer capabilities. Through a variety of signaling pathways, including X-linked inhibitors of apoptosis protein (XIAP), NF-κB, and PI3K/Akt, it prevents cell proliferation (44). In contrast to the commonly used adjuvant alum, the study discovered that luteolin can directly stimulate antigen-presenting cells (APCs) *in vitro* without antigens. This further activates the PI3K-Akt pathway, improves the CTL response, and inhibits tolerogenic T cells. Tumor development was markedly inhibited in the B16F10 mice model that received luteolin as an adjuvant. Furthermore, the study discovered that luteolin increased TH1 immunity, which may have a more significant tumor-killing effect, in comparison to the effect of alum adjuvant in stimulating TH2 immunity (45). C16H14O6 is the molecular formula for HES, a dihydroflavonoid. The B16F10 mouse model confirmed that HES can activate the PI3K-Akt signaling pathway in APC, improve CTL response. It also showed that the combination of inactivated B16F10 cells and the HES vaccine could increase the overall survival rate of mice and have a better tumor inhibition effect (46). Additionally, procyanidin (PC) boosted T cell-mediated immune responses *in vitro* and *in vivo*, stimulated CD8 T cells to release TNF-α, IFN-γ, and perforin, stopped tumor growth, and extended mouse survival in B16F10 mice (47).

#### 2.1.4 Others

Numerous uncharacterized TCM-derived agents exhibit adjuvant activity in preclinical models and warrant further investigation. For instance, in cell tests, it was discovered that Ganoderma lucidum mycelium extract (GL-m) stimulated the mRNA expression of Th1 and Th2 cytokines while inducing the proliferation of human peripheral blood mononuclear cells (PBMCs) and monocytes. By upregulating the expression of CD40, CD80, and CD86, GL-m also aided in the maturation of DCs (48). When vaccinated with ovalbumin (OVA), licorice root extract-derived nanoparticles (Glycyrrhiza-NPs) can activate antigen-specific CD8+ T lymphocytes and trigger the release of IFN-γ, which has a significant anti-tumor effect. This implies that Glycyrrhiza-NPs could develop into successful vaccine adjuvants in cancer immunotherapy (49). Gastrodin (GAS), another monomer chemical used in TCM, also demonstrated comparable promise. Gastrodin enhanced CTL effects and stimulated CD8+T cells to release cytokines, which significantly increased the immunogenicity of melanoma vaccines in both *in vitro* and *in vivo* tests (50). A helpful experiment in creating vaccine adjuvants is the combination of herbal extracts with cutting-edge technology, in addition to these individual TCM constituents. For instance, ginseng-derived extracellular vesicles-like particles (G-EVLPs) are hybridized with autologous tumor membranes to create a functional hybrid vesicle (HM-NPs). According to *in vitro* tests, HM-NPs can efficiently stimulate T lymphocyte activation by activating bone marrow-derived dendritic cells (BMDCs). According to *in vivo* tests, the HM-NPs vaccination may stimulate T cell activation and DC cell maturation in the B16F10 mouse melanoma model, and it also considerably lowers the tumor volume in mice (51). Furthermore, plant viruses exhibit promise as adjuvants for immunotherapies and vaccines of the future. Plant virus particles such as cowpea mosaic virus exhibit high immunogenicity and strong antigen adsorption capacity, along with low reactogenicity, favorable biosafety, cost-effectiveness, and scalable production (52, 53). These attributes support the exploration of plant virus particles as delivery platforms or adjuvant components in next-generation cancer vaccines.

Collectively, these findings highlight the structural and functional diversity of TCM-derived immunomodulators. However, their relative efficacy, mechanisms, and translational readiness vary substantially, warranting systematic comparison.

### 2.2 Comparison of TCM-derived vaccine adjuvants

Although diverse TCM-derived compounds show adjuvant potential, they differ markedly in clinical maturity. Among them, QS-21—derived from Quillaja saponaria—stands as the only saponin-based adjuvant approved for human vaccines, with its immunostimulatory profile validated in large-scale use. In stark contrast, polysaccharides, flavonoids, and other herbal constituents remain largely preclinical. In contrast, polysaccharides, flavonoids, and most other herbal constituents remain largely in the preclinical research phase. Among them, polysaccharides have emerged as the most extensively studied class of natural adjuvants due to their abundant sources and structural diversity (16, 54).

Regarding mechanisms of action, these adjuvant classes do not rely on a single signaling pathway but rather modulate immune responses through multi-target, synergistic interactions, with notable functional overlap. TCM-derived polysaccharides primarily engage pattern recognition receptors (PRRs)—such as TLR2/4, Dectin-1, and mannose receptor—thereby activating downstream signaling cascades including NF-κB, MAPK, and PI3K/Akt, which promote DCs maturation and drive Th1-polarized immunity (55). In comparison, QS-21 exhibits multifaceted mechanisms: its oligosaccharide moiety may bind C-type lectin receptors on antigen-presenting cells (APCs) to enhance antigen uptake, while its triterpene core induces cholesterol-dependent membrane perturbation, leading to NLRP3 inflammasome activation and subsequent release of IL-1β and IL-18—thus potently stimulating Th1 and CD8^+^ T-cell responses (56). Moreover, saponin-based adjuvants have been shown to specifically activate human conventional type 2 DCs (CD11c^+^CD1c^+^CD5^-^CD163^+^), which are highly efficient at cross-presentation (36). By contrast, flavonoids generally exert milder immunomodulatory effects, fine-tuning immune cell activity and cytokine secretion to augment overall immune function. Additionally, their potent antioxidant properties can mitigate reactive oxygen species (ROS) generation, thereby protecting immune cells from oxidative damage and preserving immune homeostasis (55).

In terms of relative potency, a systematic study in murine models comparing 19 adjuvants for a cancer vaccine containing GD3 ganglioside and MUC1 peptide demonstrated that QS-21 outperformed glucan, peptidoglycan, amphiphilic block copolymers, bacterial nucleotides, and lipopolysaccharide in eliciting both antigen-specific antibody and T-cell responses (57). Nevertheless, QS-21 faces limitations including scarcity of natural supply, poor chemical stability, and dose-dependent toxicity (57–59).

Overall, the vast majority of TCM-derived adjuvants have not yet entered human clinical trials, and their translational application confronts several critical bottlenecks. First, high-quality data on human safety and efficacy remain scarce. Second, herbal extracts are inherently complex mixtures, and their composition is highly susceptible to variations in botanical source, harvest season, and extraction protocols, resulting in poor batch-to-batch consistency. Third, systematic evaluation of potential immunotoxicity—including risks of excessive inflammation or autoimmunity—and long-term safety profiles is still inadequate. Although polysaccharides are generally considered low-toxicity and biocompatible, their capacity to induce non-specific immune activation warrants rigorous assessment using standardized models. Moving forward, establishing chemical fingerprinting, identifying marker active constituents, and implementing Good Manufacturing Practice (GMP)-compliant production will be pivotal steps toward enabling the clinical advancement of TCM-based vaccine adjuvants.

## 3 Mechanisms of TCM-derived adjuvants in tumor vaccines

In recent years, adjuvants derived from TCM have demonstrated remarkable potential in cancer immunotherapy. Their mechanisms of action span a spectrum—from direct activation of innate immune receptors and modulation of cytokine networks, to enhancement of antigen delivery via targeted carriers, and further to integrated platforms that combine both functions within a single formulation. Additionally, certain TCM compounds engage non-canonical pathways, such as saponin-induced formation of intracellular lipid bodies that promote cross-presentation. This evolving landscape reflects a shift toward multifunctional systems capable of eliciting robust and durable antitumor immunity.

### 3.1 Immunostimulatory adjuvants

#### 3.1.1 Activation of pattern recognition receptor signaling pathways

A substantial body of evidence demonstrates that plant-derived polysaccharides can activate dendritic cells (DCs) by binding to pattern recognition receptors (PRRs)—including Toll-like receptors TLR2/4 and C-type lectin receptors such as Dectin-1—thereby triggering downstream NF-κB and MAPK signaling cascades that drive DC maturation and antigen presentation. For instance, Astragalus polysaccharide (APS) has been shown to simultaneously engage TLR2, TLR4, and Dectin-1, leading to significant upregulation of the costimulatory molecule CD86 and MHC class II on bone marrow-derived DCs (BMDCs), as well as enhanced cross-presentation capacity (22). Similar mechanisms have also been reported for Pleurotus ferulae polysaccharides (60), an acidic polysaccharide from Sarcandra glabra (61), Ginseng Berry Extract (62), and high-molecular-weight polysaccharides purified from Antrodia cinnamomea (63).

Notably, certain adjuvants can concurrently activate inflammasome pathways, thereby enabling synergistic immune potentiation through multiple signaling axes. For example, Pleurotus ferulae polysaccharides conjugated to gold nanoparticles (PFPS-Au NPs) not only retain their TLR4 agonist activity but also robustly promote NLRP3 inflammasome assembly. Treatment with PFPS-Au NPs leads to time-dependent increases in the levels of cathepsin B, NLRP3, ASC, and caspase-1 (CAS-1), ultimately reinforcing DC maturation (60).

#### 3.1.2 Induction of cytokine secretion and costimulatory molecule expression

In addition to PRR activation, most traditional Chinese medicine (TCM)-derived vaccine adjuvants commonly induce DCs to secrete key cytokines—such as IL-12, TNF-α, and IL-6—and upregulate surface expression of costimulatory molecules (CD40, CD80, CD86) and MHC class I/II molecules, thereby effectively bridging innate and adaptive immunity. This phenomenon has been documented across a range of natural compounds, including a polysaccharide extracted from Grifola frondosa (64), λ-carrageenan (65), specific medicinal plant polysaccharides (66), Dioscorea phytoextracts (67), Rehmannia glutinosa polysaccharide (68), Poria cocos polysaccharide-functionalized graphene oxide nanosheets (69), procyanidin (47), and a potential herbal adjuvant combined with a peptide-based HPV vaccine (70).

For example, systemic administration of fucoidan markedly elevates IL-12 and TNF-α production in splenic conventional DCs (cDCs) and promotes the expansion of IFN-γ–producing Th1 and Tc1 cells (71). Similarly, PS-F2—a polysaccharide fraction purified from the fermentation broth of Ganoderma formosanum—stimulates DCs to secrete pro-inflammatory cytokines, including TNF-α, IL-6, and IL-12/IL-23 p40, while concomitantly upregulating maturation markers CD40, CD80, CD86, and MHC class II (72).

Flavonoids also contribute to adjuvant activity through similar mechanisms. Chrysin (CHR), a bioactive flavonoid, activates antigen-presenting cells (APCs) both *in vitro* and *in vivo* and appears to enhance Th1 responses via the IL-12–STAT4 signaling axis, thereby strengthening cytotoxic T lymphocyte (CTL)-mediated antitumor immunity in a B16F10 melanoma model (43). In another study, co-administration of luteolin with a DC–tumor fusion vaccine (delivered intravenously in a xenograft tumor model) significantly suppressed tumor growth. Luteolin treatment increased CD25 and CD69 expression on effector T cells and enhanced *in vitro* secretion of IL-2, TNF-α, and IFN-γ (73).

### 3.2 Delivery-enhancing adjuvants

An ideal tumor vaccine must not only possess strong immunogenicity but also ensure efficient delivery of antigens to secondary lymphoid organs and facilitate their uptake and processing by antigen-presenting cells (APCs). To address this challenge, several advanced delivery strategies have been developed.

One innovative approach employs ginsenoside Rg3-based lipid nanoparticles (Rg3-LNPs), which exploit the overexpression of glucose transporter GLUT1 on tumor cells for active targeting. By “tagging” tumor cells with antigen-loaded Rg3-LNPs, this system restores CTL recognition of otherwise immune-evasive tumors. Concurrently, Rg3-LNPs migrate to draining lymph nodes to activate DCs. Notably, Rg3 itself acts as an immunomodulator by inhibiting STAT3 phosphorylation, thereby reducing secretion of CCL2 and VEGF and suppressing the recruitment of M2-polarized tumor-associated macrophages (M2-TAMs)—thus achieving dual modulation of both tumor cells and the immunosuppressive tumor microenvironment (TME) (74).

Another strategy focuses on *in situ* capture of autologous tumor antigens released during cryoablation. Although cryoablation liberates a broad spectrum of tumor antigens, their rapid clearance limits immunogenicity. To overcome this limitation, researchers engineered maleimide-modified chitosan nanoparticles loaded with APS (AMNPs). These nanoparticles covalently capture diverse antigens released during cryoablation via surface-exposed functional groups and actively target lymph nodes. Importantly, AMNPs also promote endosomal escape, thereby enhancing cross-presentation efficiency. In a bilateral Lewis lung carcinoma model, combination therapy with AMNPs and cryoablation achieved 100% suppression of primary tumors, zero recurrence within 30 days, a 3.84-fold reduction in distal untreated tumor volume, and a long-term survival rate of 83.33%, underscoring its capacity to elicit robust systemic antitumor immunity (15).

### 3.3 Integrated immunostimulation and delivery adjuvants

Current trends are shifting from single-function adjuvants toward integrated “carrier–adjuvant” platforms that simultaneously deliver antigens and stimulate immunity.

For example, hybrid nanovaccines (TM-G-EVLP) constructed by fusing ginseng-derived extracellular vesicle-like particles (G-EVLPs) with autologous tumor cell membranes harvested during surgery enable personalized delivery of a patient-specific tumor antigen repertoire. Concurrently, plant-derived pathogen-associated molecular patterns (PAMPs) embedded in G-EVLPs activate DCs via TLR signaling, thereby coupling antigen delivery with innate immune stimulation (51). Similarly, an alkali-extracted arabinogalactan from Portulaca oleracea L. (POPAN), when conjugated to the model antigen BSA (POPAN-BSA), not only enhances DC uptake of the antigen but also potently induces costimulatory molecule expression and cytokine secretion, ultimately driving durable humoral and Th2-skewed cellular immune responses—demonstrating its dual functionality as both an immunopotentiator and an antigen carrier (75).

### 3.4 Additional mechanisms

Beyond the canonical pathways described above, certain TCM-derived adjuvants exert immunomodulatory effects through non-classical signaling routes or unique subcellular reprogramming, thereby expanding the mechanistic landscape of natural adjuvants.

For instance, a polysaccharide extract from Dioscorea spp. (DsCE-I) activates DCs via p38 and NF-κB pathways and significantly enhances the antitumor efficacy of a melanoma DNA vaccine. Intriguingly, NF-κB activation by DsCE-I is not fully abrogated by polymyxin B—a well-established LPS neutralizer—suggesting the involvement of TLR4-independent mechanisms, possibly mediated by alternative PRRs or intracellular sensors (27).

Furthermore, saponin-based adjuvants (SBAs) represent a distinct modality of cross-presentation regulation. SBAs selectively induce the formation of intracellular lipid bodies (LBs) in monocyte-derived CD11b^+^ DC subsets—a process closely associated with antigen routing into the proteasomal degradation pathway. Pharmacological or genetic inhibition of LB biogenesis markedly impairs SBA-driven cross-presentation and subsequent T-cell activation, establishing lipid body remodeling as a critical cellular event underlying the adjuvant activity of saponins (37).

Collectively, the field of tumor vaccine adjuvants is transitioning from traditional single-mode immune activators toward multifunctional, self-adjuvanting systems that integrate antigen delivery with coordinated immune stimulation. A deeper mechanistic understanding of how these adjuvants modulate APC function, key signaling nodes (e.g., TLR/NF-κB/MAPK/NLRP3), and T-cell differentiation will not only elucidate the scientific basis of TCM-derived compounds but also provide essential insights for the rational design of next-generation cancer vaccines.

## 4 The forms of TCM-derived adjuvants in tumor vaccines

We have summarized the components and mechanisms used in TCM as adjuvants. Optimized formulations enhance vaccine controllability and precision in delivery (76, 77). This section mainly summarized the forms, advantages, and safety of TCM as adjuvants based on existing research to develop more effective adjuvants for tumor vaccines.

### 4.1 Polymer nanoparticles

Polymer nanoparticle delivery systems can improve the immunogenicity of antigens, minimize toxicity, enhance antigen uptake and entry into the cell nucleus, and improve the overall antigen-specific immune response. Commonly used ones include chitosan, polylactic acid (PLA), polyglutamic acid (PGA), and polylactic-co-glycolic acid copolymer (PLGA) (78).

PLGA is a biocompatible and biodegradable polymer characterized by excellent biocompatibility and tunable degradation kinetics. Approved by the U.S. Food and Drug Administration (FDA) for vaccine delivery applications, PLGA exhibits superior drug encapsulation efficiency and controlled-release properties (79). Several studies have encapsulated biological components from the TCM into PLGA as adjuvants, including Alhagi honey polysaccharides (80) and Angelica sinensis polysaccharides (23). The antigen is continuously and controllably released *in vivo*, significantly improving the CD4 +/CD8 + T cell ratio and increasing the IgG level, upregulating cytokines, and leading to overall Th1 polarization.

Chitosan is a naturally occurring polymer with cationic ions on its surface. When dissolved in acidic media, the amino group of chitosan becomes highly protonated, allowing electrostatic interactions between chitosan and mucins, resulting in solid adhesion properties for chitosan. Numerous chitosan-based derivatives, including nano-chitosan, have been developed for the encapsulation of drugs and vaccines. Owing to their excellent biocompatibility and biodegradability, nanoscale chitosan derivatives are ideal candidates for biomedical applications, such as drug delivery and tissue engineering. However, particle size, surface charge, and chemical modifications may influence their biodistribution and cytotoxicity. Future research will require comprehensive *in vivo* studies to evaluate long-term safety profiles (81). Maleimide-modified Pluronic F127-chitosan nanoparticles encapsulating Astragalus polysaccharide (AMNPs) are developed. AMNPs are capable of sequestering a wide array of immunogenic tumor antigens released as a result of cryoablation. They possess an inherent ability to direct themselves toward lymph nodes and facilitate the lysosomal release, which is pivotal for activating distant DCs. This process further mediates T cell differentiation via cross-presentation mechanisms. Ultimately, this strategy disrupts the tumor’s immunosuppressive microenvironment, leading to the establishment of enduring and potent tumor-specific immune responses (15).

### 4.2 Liposomes

Liposomes are closed vesicles composed of lipid bilayers of phospholipids and cholesterol, with biological membrane-like structures encapsulating water-soluble and lipid-soluble drugs. Liposomes represent the most extensively utilized vaccine delivery systems in clinical settings, owing to their biocompatibility, biodegradability, and nontoxic properties. They have received approval from the FDA for human use. They are often used as drug carriers and have many advantages, such as high targeting specificity, adequate protection of encapsulated drugs, controlled and gradual release of drugs, improved therapeutic index, and reduced side effects (82, 83). Liposomes have been extensively studied as delivery systems for ingredients, including those derived from herbal Medicine. Encapsulated with herbal active ingredients for Rehmannia glutinosa polysaccharide (16), Lycium barbarum polysaccharides (84), Ganoderma lucidum polysaccharide (85), and epimedium polysaccharide (20), can be used as a suitable adjuvant. Equally important is the fact that lipid nanoparticles (LNPs) are not merely delivery vehicles; they inherently possess adjuvant activity. The combination of LNPs with modified mRNA has been shown to induce innate immune signaling essential for driving both human T-cell and B-cell responses. For instance, LNPs can stimulate immune cells to release IL-6. This effect may be linked to the recognition of either the whole LNP or its ionizable lipid component by one or more pattern recognition receptors (PRRs). This adjuvant activity may also be responsible for the fever and pain observed with clinical influenza vaccines (86).

### 4.3 Pickering emulsion

Pickering emulsion was designed by Yufei Xia et al. based on the dynamic bending and lateral diffusion of the membrane when the antigen is internalized by immune cells. It can increase the contact area and multivalent interaction with antigen-presenting cells and has been proven to be an effective vaccine delivery system (87, 88). The primary distinction from conventional surfactant-stabilized emulsions lies in the absence of surfactants in this system. Instead, nanoparticles possessing an amphiphilic nature (exhibiting both hydrophilic and lipophilic properties) are employed as emulsion stabilizers. By adjusting the properties of the particles and the parameters of the oil and aqueous phases, particle-stabilized emulsions were obtained via ultrasonication. Pickering emulsions offer advantages, including being surfactant-free, exhibiting low toxicity, and demonstrating high stability (89). Studies have shown that Pickering emulsions containing Chinese yam polysaccharides, PLGA-stabilized Pickering emulsion (90), Poria cocos polysaccharide (91), and Lycium barbarum polysaccharide (84) can induce strong and long-lasting immune responses can act as adjuvants to enhance humoral and cellular immunity.

### 4.4 Exosomes

Exosomes are emerging as a promising carrier for nucleic acid vaccine delivery (76, 92, 93). These exosomes are 40-150nm in diameter, stable, safe, and have low immunogenicity (79). The exosomes were initially described as a cell-to-cell communication mechanism in eukaryotes. Subsequently, scientists found that this communication mechanism is widely present in various life forms, including prokaryotes and plants. Interestingly, extracellular vesicles can mediate the horizontal transfer of nucleic acids, and their lipid bilayer membrane can protect nucleic acids from degradation (94).

Recent studies have developed a nanotherapeutic drug delivery systems using fused nanovesicles from plant and animal cells with high clinical potential. Haoran Wang et al. used ginseng-derived extracellular vesicle-like particles to hybridize with membranes from resected autologous tumors to form functional hybrid vesicles and found that they enhanced the phagocytosis of autologous tumor antigens by DCs, promoted DCs maturation through Toll-like receptor 4(TLR4), and ultimately activated tumor-specific CTLs (51). Nanovesicles derived from TCM is rich in sRNA, small molecule compounds, lipids, protein peptides, plant fibers, polysaccharides, and various elements. They are exosome-like nanoparticles; their substances are the active ingredients of TCM decoctions. Existing studies have extracted exosome-like vesicles from edible plant (citrus) juice as carriers, loaded the mRNA encoding SARS-CoV-2 into the exosomes, lyophilized and encapsulated in enteric-coated capsules, and then orally administered to rats. The results showed that compared with synthetic nanoparticles, exosomes were more easily internalized by cells, promoting the production of IgM, IgG, and IgA in rats (95). The TCM can self-assemble to generate new nano-like vesicles during the decoction process (96–99), and the multi-component characteristics are TCM for the individualized treatment of cancer. Exosome-like vesicles extracted from TCM can potentially be tumor vaccine adjuvants. However, research in this area remains preliminary. The safety profiles of herbal-derived extracellular vesicles (EVs) when utilized as vaccine adjuvants warrant further rigorous investigation.

### 4.5 Sporopollenin

People have explored many natural and synthetic materials to prepare antigen carriers, but there are problems, such as low potential toxicity and biodegradability in body fluids. In recent years, people have begun to study sporopollenin as a delivery system (100–102) to solve these problems. Sporopollenin is the general term for spores of spore plants and pollen of seed plants. It has a natural core-shell structure and exquisite surface morphology. They are relatively uniform in size; most have an inner cavity, have suitable porosity and adhesion, and can be used as natural drug delivery carriers (103). Jun Liu et al. used sporopollenin powder from wild chrysanthemum as an adjuvant, loaded it with antigens, and administered it through the nasal mucosa. They eventually found that the w/o/w emulsion with the chrysanthemum sporopollenin vaccine delivery system can induce secretory IgA antibodies in the nasal mucosa. Compared with squalene emulsion adjuvants, it stays in the nasal mucosa longer. It can produce stronger humoral responses (IgA and IgG) and demonstrate a favorable safety profile (104). Another study is that Astragaloside IV (AS-IV), the main bioactive ingredient of Astragalus, can be made into mesoporous and excipient-free microfibers (SMF), which spontaneously assemble into a scaffold with a macroporous 3D structure after injection *in vivo*, recruiting a large number of DCs into the macropores between the stacked fibers and enhancing the maturation of DCs through the NLRP3 pathway (105). They represent a promising platform technology for developing safe and effective tumor vaccines through advanced immune modulation, though their safety profile remains uncharacterized. Future research in tumor vaccine development should prioritize comprehensive safety evaluation.

### 4.6 Chemical modification

Chemical modification can change the structure of polysaccharides, thereby improving their performance or giving them new functions (106), including selenization (107), sulfation (108), phosphorylation (109), etc. Haibo Feng et al. used the HNO3 -Na2SeO3 method to couple Chuanminshen violaceum polysaccharides (CVPS) with selenium to synthesize selenium-modified CVPS (sCVPS). They found that sCVPS as an adjuvant can enhance the activity of NK cells and CTLs, thereby strengthening the cellular and humoral immune responses (110). QS-21, a natural saponin product, is the preferred immunopotentiator in many clinical cancer and infectious disease vaccine trials. However, it is scarce, difficult to purify to homogeneity, and has dose-limiting toxicity and chemical instability. The entire branched trisaccharide domain structure is removed or the structure is adjusted by some methods, such as stepwise truncation of the linear tetrasaccharide Domain, to reduce its toxicity (111).

### 4.7 Self-assembled peptides

Commonly used carriers have toxicity and antigen carrier density problems in the body. There are also studies on the self-assembly of antigens and adjuvants into nanoparticles to exert their effects on the body. Astragalus polysaccharide, as a drug carrier and immune adjuvant, forms nanocomplexes with peptide antigens through microfluidics, which can resist tumor adaptive immune response with no observed systemic side effects (22). Another study mixed Lentinan with ovalbumin (OVA) to obtain self-assembled nanovaccines to promote the uptake and maturation of BMDCs and the response of helper T lymphocytes with no significant systemic toxicity, demonstrating favorable biocompatibility, thereby supporting its utility as a safe adjuvant platform (112).

### 4.8 Silica nanoparticles

Among inorganic nanomaterials, silica nanoparticles have the characteristics of high surface area, bioconjugation, biocompatibility, size and pore size adjustability, multifunctional surface chemistry, and high loading efficiency, and have demonstrated potential for biomedical applications (113). Nanoporous silica particles function dually as both antigen delivery vehicles and immunostimulants. They modulate cellular and humoral immune responses by enhancing antigen presentation and stimulating immune reactions. Ghasemi and colleagues investigated the subchronic (180 day) and chronic toxicity of these particles using animal models. *In vitro* analyses indicated low subchronic toxicity for mesoporous silica nanoparticles (MSNs), with no observable significant harm to animals, absence of hematotoxicity, and no complement system activation (114). Compared with traditional silica nanoparticles, the dendritic fibrous nano-silica (DFNS) has attracted much attention due to its unique open three-dimensional dendritic superstructure, larger pore size, and internal surface area. Cistanche deserticola polysaccharide (CDP) has multiple biological activities, such as antioxidant, liver protection, promotion of immune cell proliferation, regulation of intestinal flora, and activation of DCs (115–117). Jin He et al. synthesized the DFNS grafted with CDP nanoparticles as an adjuvant for oral vaccines, inducing strong systemic and mucosal antibody responses (118).

### 4.9 Direct injection

Ingredients derived from TCM, such as polysaccharides, can be directly injected into the body to stimulate the proliferation of immune cells. For example, Danping Zhao et al. injected Astragalus polysaccharides (APS) intramuscularly into BALB/c mice infected with the influenza virus. The results showed that APS is a potential adjuvant for enhancing vaccine immune response, with the advantages of bidirectional immunomodulation and persistent immunity (119). Minseok Kwak et al. injected Rehmannia glutinosa polysaccharide (RGP) intranasally into C57BL/6 mice and found that it can stimulate DC and T cells and release cytokines. Using RGP as a mucosal adjuvant and in combination with tyrosinase-related protein 2 (TRP2) peptide as an antigen of melanoma cells showed better therapeutic effects (68).

## 5 Potential for clinical application

Herbal medicines have demonstrated immunomodulatory activity that warrants their investigation as adjuvants in tumor immunotherapy. Evidence indicates that TCM formulations can alleviate immunosuppression within the tumor microenvironment, enhance dendritic cell and macrophage activation, improve tumor antigen presentation, and promote T-cell proliferation and infiltration—thereby counteracting tumor immune evasion (120). Moreover, certain herb-derived compounds exhibit adjuvant-like activity, particularly when formulated into nanocarriers that enhance bioavailability and enable more precise targeting of immune cells (121). Despite these mechanistic insights, no TCM-derived adjuvant has yet advanced to late-stage clinical trials for cancer vaccines, highlighting a significant gap between preclinical promise and clinical translation.

This translational bottleneck stems largely from fundamental challenges in standardization, regulatory compliance, and safety assessment. In contrast to established vaccine adjuvants—such as QS-21, aluminum salts, AS01, and AS03—which are chemically well-defined and manufactured under stringent Good Manufacturing Practice (GMP) conditions, most TCM-based candidates remain complex, multicomponent mixtures with inherent batch-to-batch variability. Factors including plant species, geographic origin, harvest time, extraction methodology, and storage conditions profoundly influence their chemical composition and biological activity.

Compounding this issue is the notable lack of human safety and reactogenicity data. While veterinary studies offer preliminary support—for instance, compound Chinese herbal medicinal ingredients (cCHMIs) containing astragalus polysaccharide (APS), epimedium polysaccharide (EPS), propolis flavonoids (PF), and ginsenosides (GS) enhanced lymphocyte proliferation and IFN-γ/IL-10 mRNA expression in rabbits vaccinated against rabbit hemorrhagic disease virus (122)—these findings cannot substitute for controlled human trials. Even formulations with robust antitumor efficacy in murine models, such as Gan Sui Bian Xia Tang (GSBXD), which reduced myeloid-derived suppressor cell (MDSC) accumulation and modulated CD3^+^NK1.1^+^ NK-cell ratios via inhibition of the AKT/STAT3/ERK pathway in H22 tumor-bearing mice (123), lack the GLP-compliant toxicology, dose-ranging, and immunotoxicity profiles required to support an Investigational New Drug (IND) application. Without such data, concerns regarding potential off-target immune activation, cytokine dysregulation remain unresolved.

Notably, Advax™ delta—a clinically validated adjuvant composed of delta inulin, a polysaccharide also found in TCM herbs such as Codonopsis pilosula, Arctium lappa, and Atractylodes macrocephala (124–126)—provides a rare proof-of-concept that plant-derived molecules can meet modern regulatory standards. Evaluated in multiple Phase I–III trials for influenza and other vaccines, Advax™ delta demonstrated acceptable safety, enhanced immunogenicity even at antigen-sparing doses (127), and promoted B-cell affinity maturation through mechanisms including AID upregulation and CDR3 mutagenesis (128). Critically, its reactogenicity profile was mild and transient, with no serious adverse events attributed to the adjuvant itself. Beyond infectious disease applications, inulin has also shown intrinsic antitumor activity in preclinical models, suppressing colitis-associated colon carcinogenesis (129) and enhancing Th1-polarized CD4^+^/CD8^+^ T-cell responses across multiple murine tumor systems (130). Nevertheless, these anticancer effects remain unvalidated in human trials, and the success of Advax™ delta hinges on its status as a purified, structurally defined polysaccharide—a stark contrast to crude or multi-herb TCM extracts.

Moving forward, realizing the clinical potential of TCM in tumor vaccine adjuvant development will require a systematic, de-risking strategy. Priority should be given to the isolation and GMP-scale production of single, well-characterized active monomers (e.g., APS, specific ginsenosides); standardized fingerprinting of complex formulations using metabolomics coupled with bioassay-guided fractionation; comprehensive GLP-compliant toxicology and immunotoxicity assessments; and, ultimately, early-phase clinical trials with rigorous monitoring of reactogenicity and immune-related adverse events. Only through such disciplined integration of traditional knowledge with modern pharmaceutical standards can select TCM-derived agents transition from empirical remedies to rationally designed, regulatory-compliant components of next-generation cancer immunotherapies.

## 6 Discussion

Cancer, as a complex systemic chronic disease, severely threatens human health and quality of life, with immunologically “cold tumors” posing a major clinical challenge due to their lack of effective T-cell infiltration and immune-activating signals. Tumor vaccines show translational potential for remodeling the cold tumor microenvironment by delivering tumor antigens to activate antigen-specific immune responses, where adjuvants as core vaccine components critically determine immunological success. The proven efficacy of clinically adopted plant-derived adjuvants (e.g., QS-21) (131, 132), and the rich repository of immunomodulatory compounds in Chinese herbal medicine (such as polysaccharides, saponins, and flavonoids) (133–135), establishes a scientific foundation for developing vaccine adjuvants with Chinese characteristics. This review systematically examines the active components, mechanisms, and forms of Chinese herbal-derived adjuvants to accelerate their translation from bench to bedside.

Presently, only seven adjuvants are approved for human vaccines (aluminum hydroxide, MF59, AS01, virosomes, AS03, AS04, and CpG 1018), each typically activating either cellular or humoral immunity (16). In contrast, herbal components uniquely enable dual-pathway activation of both cellular and humoral immunity, crucial for tumor cell clearance, while offering advantages including high safety, enhanced drug tolerability, potentiated humoral/cellular immunity, negligible toxicity, minimal side effects, and broad applicability (136).

However, bottlenecks persist: the compositional complexity of herbal formulas complicates quality control and obscures active constituents; research remains confined to murine models with critical gaps in human pharmacokinetics and dose-response relationships, hindering clinical translation; the immunosuppressive tumor microenvironment may be limits vaccine efficacy, future combinations with immune checkpoint inhibitors (e.g., anti-PD-1/PD-L1); and regulatory barriers in Western agencies, where “unclear composition implies uncontrollability”, demand novel evaluation paradigms.

Addressing these challenges requires focused strategies: deepening research through the “sovereign-minister-assistant-courier” compatibility principle to identify synergistic combinations, employing high-resolution mass spectrometry and molecular docking to pinpoint key active components, accelerating clinical translation of herbal adjuvants for tumor vaccine development, and pioneering combination therapies with immune checkpoint inhibitors or CAR-T cells to synergistically enhance anti-tumor immunity, overcome immunosuppressive microenvironments, and improve therapeutic durability. Collectively, TCM-derived adjuvants may represent a viable strategy for tumor vaccines, poised to evolve from empirical tradition to evidence-based medicine through systematic constituent elucidation and precision combination strategies.

## 7 Conclusions and future perspectives

Polysaccharides, saponins, and flavonoids derived from Chinese herbal medicines have been extensively investigated as adjuvants for cancer vaccines. Delivered via advanced systems such as liposomes, polymeric nanoparticles, and exosomes, these compounds not only enhance antigen uptake and presentation *in vivo* but also potently augment T-cell activation and tumor-killing capacity, demonstrating significant potential as immunological adjuvants. Traditional Chinese medicine (TCM) and its bioactive constituents offer unique advantages in modulating dendritic cell maturation, promoting Th1/CTL responses, and reshaping the immunosuppressive tumor microenvironment, underscoring their substantial value for further exploration as vaccine adjuvants.

Looking toward clinical translation, future efforts should prioritize two key directions. First, strategic integration of TCM-derived adjuvants with cutting-edge immunotherapies. Given that monotherapy with cancer vaccines often fails to overcome the immunosuppressive tumor milieu, combining them with immune checkpoint inhibitors (e.g., anti–PD-1/PD-L1 antibodies) (137) may help reverse T-cell exhaustion. Additionally, TCM adjuvants could complement chimeric antigen receptor T-cell (CAR-T) therapy by improving tumor antigen visibility or enhancing T-cell infiltration, thereby boosting efficacy against solid tumors.

Second, leveraging computational drug design to accelerate the discovery of novel adjuvants (138–140). By integrating TCM compound databases with molecular docking, network pharmacology, and artificial intelligence models, researchers can efficiently identify candidate molecules with pattern recognition receptor (PRR)-agonistic activity or dendritic cell–activating potential. This approach enables a paradigm shift—from empirical use toward rational, precision-driven adjuvant design.

Through interdisciplinary convergence and deeper mechanistic understanding, TCM shows potential to deliver safe, effective solutions for next-generation cancer vaccine adjuvants.
